# Risk factors associated with postoperative respiratory failure in tuberculous empyema patients

**DOI:** 10.1097/MD.0000000000025754

**Published:** 2021-06-11

**Authors:** Hongyun Ruan, FangChao Liu, Changfan Gong, Xinting Yang, Ming Han

**Affiliations:** a Cardiopulmonary Function Department; b Science and Technology Office; c Department of Thoracic Surgery; d Department of Tuberculosis, Beijing Chest Hospital, Capital Medical University/Beijing Tuberculosis and Thoracic Tumor Research Institute, Beijing, PR China.

**Keywords:** postoperative respiratory failure, surgical treatment, tuberculous empyema

## Abstract

Our objective was to identify independent risk factors for predicting which patients in the Chinese population would likely develop respiratory failure.

A descriptive analysis was conducted of demographic and clinical data of patients with tuberculous empyema (TE) admitted to the Beijing Chest Hospital, Capital Medical University between January 2001 and January 2020. Risk factors associated with postsurgical respiratory failure in TE patients were identified based on results of analyses based on univariable and multivariable logistic regression models.

A total of 139 TE patients who underwent surgical treatment in the Beijing Chest Hospital, Capital Medical University from January 2001 to January 2020 were enrolled in this study. Cases included 109 male and 30 female patients, with an overall mean age (range 17–73) of 39.3 years. Of 139 TE patients, 26 (18.7%) experienced respiratory failure after surgery. Among significant risk factors for postsurgical respiratory failure, intraoperative blood loss volume greater than 1000 mL had the highest odds ratio value of 6.452. In addition, a pathologic preoperative pulmonary function test result showing a high partial pressure of carbon dioxide level was an independent risk factor for respiratory failure. Moreover, the presence of tuberculosis lesions in the contralateral lung was another significant risk factor for respiratory failure, as determined using multivariate analysis.

Respiratory failure is a predominant complication experienced by TE patients undergoing surgery. High intraoperative blood loss, high preoperative high partial pressure of carbon dioxide level, and tuberculosis lesion(s) in the contralateral lung of TE patients were associated with increased risk of postoperative respiratory failure.

## 1 Introduction

Tuberculosis (TB), caused by *Mycobacterium tuberculosis* (MTB), remains a major public health concern worldwide.^[1]^ MTB typically affects the lungs but can also affect any site outside the lungs, resulting in extrapulmonary tuberculosis (EPTB).^[2,3]^ In past decades, multiple studies conducted in wealthy countries have demonstrated that EPTB cases comprise an increasing proportion of total TB cases, underscoring EPTB as an urgent global health challenge.^[3,4]^ Pleural TB, one of the most frequently diagnosed forms of EPTB, accounted for approximately 30% to 40% of all EPTB cases in a previous report.^[5]^ Although the majority of pleural TB patients are cured after treatment with standardized anti-TB regimens, a small proportion of patients progress to tuberculosis empyema (TE) in spite of medical treatment,^[6]^ further leading to formation of chronic and fatal sequelae.^[7]^

After a pleural TB infection responds poorly to chemotherapy, surgical intervention is considered the most potent treatment approach for avoiding subsequent development of chronic empyema.^[8]^ Although it might be natural to anticipate that surgically treated patients would have favorable treatment outcomes, several investigators have reported dramatically high surgery-associated mortality rates in various populations,^[9,10]^ with postsurgical respiratory failure pinpointed as a major risk factor for mortality.^[10]^ Therefore, early interventions undertaken to protect individuals at high risk of postsurgical respiratory failure is essential to reduce the death rate. Unfortunately, limited knowledge has hampered timely delivery of preventive measures. To address this concern, we conducted a retrospective study of tuberculous empyema patients undergoing surgery between January 2001 and January 2020. Our objective was to identify independent risk factors that could be used to predict which patients within the overall Chinese population were most likely to develop respiratory failure.

## 2 Materials and methods

### 2.1 Data sources and collection

A descriptive analysis was performed to assess risk factors for postsurgical respiratory failure based on demographic and clinical data obtained for TE patients undergoing treatment at the Beijing Chest Hospital, Capital Medical University between January 2001 and January 2020. Beijing Chest Hospital, Capital Medical University is a 653-bed hospital that delivers specialized therapy for TB and thoracic diseases.^[2]^ All TE patients undergoing surgery were included in our analysis except for those who were afflicted with respiratory failure before surgery or those who lacked complete medical records. This study was approved by the Ethics Committee of Beijing Chest Hospital, Capital Medical University.

We routinely conducted various examinations on all patients suspected of having TE, including acid-fast bacilli (AFB) smear- and culture-based testing, molecular diagnostic testing of sputum and pleural fluid, and histological examination of pleural tissue. A diagnosis of TE was made based on any of the following diagnostic criteria:

(1) Positive AFB staining of pleural effusion or tissue specimen;
(2) Positive MTB pleural effusion or tissue culture result;
(3) A pathological biopsy result for a pleural specimen revealing granulomatous inflammation with caseous necrosis or positive AFB after examination of a histopathological tissue section (after ruling out nontuberculous mycobacterial infection).

Baseline characteristics, such as sociodemographic data, comorbidities, and clinical variables, were collected from patient medical histories stored in an electronic patient record system. Respiratory failure was diagnosed for patients while they inhaled room air if they had the following signs: partial pressure of oxygen <60 mm Hg (1 mm Hg = 0.133 kPa) and/or partial pressure of carbon dioxide (PaCO_2_:) >50 mm Hg, while also exhibiting significant clinical symptoms of respiratory failure.^[11,12]^ According to World Health Organization's definitions, individuals were categorized based on body mass index (BMI) into 3 categories: (BMI) <18.5 kg/m^2^ (underweight), BMI 18.5 to 24.9 (normal weight), and BMI ≥25 kg/m^2^ (overweight).^[13]^ Meanwhile, individuals with smoking index values >400 were defined as heavy smokers. Mycobacterial isolates obtained from all TB cases with positive mycobacterial cultures were examined for in vitro drug susceptibility. Patients infected with MTB with resistance to both rifampin and isoniazid were defined as multidrug-resistance TB cases.^[14]^ The following lung function parameters were assessed: vital capacity as a percentage of expected value of vital capacity, deep inspiratory volume as a percentage of expected value of deep inspiratory volume, residual gas volume as a percentage of expected residual gas volume value, forced vital capacity, forced expiratory volume in 1 second, forced expiratory volume and forced vital capacity in the first second, maximum minute ventilation, peak expiratory flow, total airway resistance, diffusing capacity, and instantaneous expiratory flow in force of 50% vital capacity. Lung function parameter percentages <80% of expected values were judged to be decreased based on pulmonary function testing standards issued by the European Respiratory Society. Laboratory examinations were performed 24 to 60 hours before surgeries.

### 2.2 Statistical analysis

The mean and standard deviation were calculated for normally distributed continuous variables, while numerical data and proportional data were tabulated for categorical variables. Factors associated with postsurgical respiratory failure in TE patients were determined using univariable and multivariable logistic regression models. Forward stepwise multivariate models were built after inclusion of variables with *P *< .05. All calculations were conducted using SPSS version 21.0 for Windows (SPSS Inc., Chicago, IL). A *P*-value less than .05 obtained after statistical analysis was considered statistically significant.

## 3 Results

### 3.1 Patients

A total of 139 TE patients undergoing surgery in the Beijing Chest Hospital, Capital Medical University between January 2001 and January 2020 were included in this study. Cases included 109 male and 30 female patients with an overall mean age (range 17–73) of 39.3 years old. Of these study subjects, 48 (34.5%) had a history of smoking, of whom 10 (10/48, 20.8%) were heavy smokers with smoking index ≥400. Comorbidities were noted in 15 patients, for an overall patient comorbidity rate of 10.8%. In addition, before surgery, 121 (87.1%) patients received anti-TB medications, including 115 (82.7%) who received first-line drugs and 6 (4.3%) who received second-line drugs. Of 139 TE patients, 26 (18.7%) experienced respiratory failure after surgery (Table 1).

**Table 1:** Univariate analysis of demographic and clinical characteristics of TE patients stratified to the presence of respiratory failure.

Variables	Total (n = 139, %)	Nonrespiratory failure group (n = 113, %)	Respiratory failure group (n = 26, %)	OR (95% CI)	*P*-value
Sex					
Male	109 (78.4)	86 (78.9)	23 (21.1)	2.407 (0.670–8.644)	.178
Female	30 (21.6)	27 (90.0)	3 (10.0)	Ref.	
Age group (years)					
≤65	130 (93.5)	106 (81.5)	24 (18.5)	Ref	
>66	9 ( 6.5)	7 (77.8)	2 (22.2)	1.262 (0.247–6.458)	.780
BMI (kg/m^2^)					
<18.5	20 (14.4)	16 (80.0)	4 (20.0)	0.969 (0.284–3.300)	.960
18.5–24.9	78 (56.1)	62 (79.5)	16 (20.5)	Ref	.729
≥25.0	41 (29.5)	35 (85.4)	6 (14.6)	0.664 (0.238–1.853)	.434
Smoking history					
No	91 (65.5)	73 (81.1)	17 (18.9)	Ref	
Yes	48 (34.5)	40 (81.6)	9 (18.4)	0.966 (0.395–2.365)	.940
Smoking index					
≤400	38 (79.2)	32 (84.2)	6 (15.8)	Ref	
>400	10 (20.8)	7 (70.0)	3 (30.0)	2.286 (0.457–11.426)	.314
Alcohol abuse					
No	124 (89.2)	100 (80.6)	24 (19.4)	Ref	
Yes	15 (10.8)	13 (86.7)	2 (13.3)	0.641 (0.136–3.032)	.575
Comorbidities^∗^					
No	124 (89.2)	101 (81.5)	23 (18.5)	Ref	
Diabetes^†^	8 (5.8)	6 (75.0)	2 (25.0)	1.464 (0.277–7.723)	.653
Hypertension^†^	5 (3.6)	4 (80.0)	1 (20.0)	1.098 (0.117–10.288)	.935
Coronary artery disease^†^	2 (1.4)	2 (100.0)	0 (0.0)	–	.999
Yes	15 (10.8)	12 (80.0)	3 (20.0)	1.098 (0.286–4.208)	.82
Course of disease					
≤120 mo	124 (89.2)	103 (91.8)	21 (8.2)	Ref	
>120 mo	15 (10.8)	10 (66.7)	5 (33.3)	1.566 (0.872–2.813)	.133
Site of TE					
Left lung	76 (54.7)	64 (84.2)	12 (15.8)	Ref	
Right lung	63 (45.3)	49 (77.8)	14 (22.2)	1.524 (0.647–3.587)	.335
Anti-TB medication					
No	18 (12.9)	13 (72.2)	5 (27.8)	Ref	.578
First-line drugs	115 (82.7)	95 (82.6)	20 (17.4)	0.547 (0.175–1.709)	.300
Second-line drugs	6 (4.3)	5 (83.3)	1 (16.7)	0.520 (0.048–5.629)	.591
MDR					
No	135 (97.1)	110 (81.5)	25 (18.5)	Ref	
Yes	4 (2.9)	3 (75.0)	1 (25.0)	1.467 (0.146–14.694)	.745
TB lesion in contralateral lung					
No	90 (64.7)	79 (87.8)	11 (12.2)	Ref	
Yes	49 (35.3)	34 (69.4)	15 (30.6)	3.168 (1.320–7.606)	.010

BMI = body mass index, CI = confidence interval, MDR = multidrug resistance, OR = odds ratio, TE = tuberculous empyema.

∗ Comorbidities include diabetes, hypertension, coronary artery disease. In view of small sample size of patients with comorbidities, they are combined into 1 group for statistical analysis.

† Due to insufficient sample size, Fisher exact test was used.

### 3.2 Demographic and clinical characteristics of TE patients

We first analyzed demographic and clinical characteristics of TE patients stratified according to occurrence of respiratory failure. Results of bivariate analysis demonstrated that male patients were more likely to experience respiratory failure after surgery. Based on control group patients with BMI values of 18.5 to 24.9 kg/m^2^, respiratory failure was less likely experienced by patients with BMI values greater than 18.5 kg/m^2^. Meanwhile, patients with TB history were shown to be at greater risk for respiratory failure as compared to patients without TB history. Patients with TB lesions in the contralateral lung were at significantly higher risk of experiencing respiratory failure as compared to patients without TB lesions in the contralateral lung (Table 2).

**Table 2:** Univariate analysis of laboratory and clinical examinations of TE patients stratified to the presence of respiratory failure.

Variables	Total (n = 139, %)	Nonrespiratory failure group (n = 113, %)	Respiratory failure group (n = 26, %)	OR (95% CI)	*P*-value
Lesion size					
≤7 cm	16 (11.5)	15 (93.8)	1 (6.2)	Ref	
>7 cm	123 (88.5)	98 (80.0)	25 (20.0)	3.827 (0.482–30.365)	.204
Mediastinal shift					
No	89 (64.0)	74 (83.1)	15 (16.9)	Ref	
Yes	50 (36.0)	39 (78.0)	11 (22.0)	1.391 (0.583–3.319)	.456
Pulmonary function					
VC%pred		67.0 ± 14.3	63.3 ± 11.5	0.980 (0.949–1.012)	.218
IC%pred		61.9 ± 19.5	60.9 ± 14.3	0.997 (0.974–1.021)	.841
RV%pred		102.6 ± 28.4	109.0 ± 28.8	1.008 (0.993–1.022)	.299
FVC%pred		68.7 ± 15.1	64.7 ± 12.2	0.981 (0.952–1.011)	.213
FEV_1_%pred		69.3 ± 17.2	63.4 ± 13.6	0.979 (0.954–1.005)	.109
FEV_1_/FVC		84.0 ± 10.6	79.7 ± 11.2	0.965 (0.928–1.003)	.071
FEF50%pred		68.8 ± 29.7	54.6 ± 24.2	0.982 (0.967–0.998)	.028
MVV%pred		74.1 ± 23.9	68.7 ± 15.5	0.989 (0.970–1.009)	.278
PEF%pred		71.2 ± 20.6	63.5 ± 19.8	0.982 (0.962–1.003)	.086
DLCO%pred		71.1 ± 13.7	72.7 ± 15.2	1.008 (0.978–1.039)	.599
Electrocardiogram					
Normal	104 (74.8)	86 (82.7)	18 (17.3)	Ref	
Abnormal	35 (25.2)	27 (82.9)	8 (17.1)	1.416 (0.554–3.618)	.468
Leukocyte					
Normal	137 (98.6)	112 (81.8)	25 (18.2)	Ref	
Increased	2 (1.4)	1 (50.0)	1 (50.0)	4.480 (0.271–74.079)	.295
Creatinine					
Normal	131 (94.2)	107 (81.7)	24 (18.3)	Ref	
Decreased	8 (5.8)	7 (87.5)	1 (12.5)	0.637 (0.075–5.422)	.680
CRP					
Normal	70 (50.4)	53 (80.0)	17 (20.0)	Ref	
Increased	69 (49.4)	60 (84.1)	9 (15.9)	0.468 (0.192–1.137)	.094
Platelet					
Normal	134 (96.4)	110 (82.1)	24 (17.9)	Ref	
Decreased	5 (3.6)	4 (80.0)	1 (20.0)	1.146 (0.123–10.713)	.905
ESR					
Normal	120 (86.3)	100 (68.3)	20 (16.7)	Ref	
increased	19 (13.7)	14 (73.7)	5 (26.3)	1.786 (0.578–5.519)	.314
Albumin					
Normal	81 (58.3)	69 (85.2)	12 (14.8)	Ref	
Decreased	58 (41.7)	45 (77.6)	13 (22.4)	1.661 (0.696–3.964)	.253
Hemoglobin					
Normal	120 (86.3)	101 (84.2)	19 (15.8)	Ref	
Decreased	19 (13.7)	13 (68.4)	6 (31.6)	3.101 (1.082–8.889)	.035
Blood glucose					
Normal	109 (78.4)	92 (84.4)	17 (15.6)	Ref	
Increased	30 (21.6)	22 (73.3)	8 (26.7)	1.968 (0.753–5.142)	.167
pH					
Normal	124 (89.2)	104 (92.0)	20 (16.2)	Ref	
Increased	15 (10.8)	9 (60.0)	6 (40.0)	0.971 (0.410–2.302)	.947
PaO_2_ (mm Hg)					
Normal	116 (83.5)	92 (79.3)	24 (20.7)	Ref	
Decreased	23 (16.5)	22 (95.7)	1 (4.3)	5.739 (0.736–44.748)	.095
PaCO_2_ (mm Hg)					
Normal	112 (80.6)	96 (85.7)	16 (15.2)	Ref	
Increased	27 (19.4)	17 (63.0)	10 (37.0)	3.529 (1.374–9.067)	.009

CI = confidence interval, CRP = C-reaction protein, DLCO%perd = diffusing capacity, ESR = erythrocyte sedimentation rate, FEF50% = instantaneous expiratory flow in force of 50% vital capacity, MDR = multidrug resistance, MVV%perd = maximum minute ventilation, PaCO_2_ = partial pressure of carbon dioxide, PaO_2_ = partial pressure of oxygen, PEF%perd = peak expiratory flow, TE = tuberculous empyema.

### 3.3 Laboratory and clinical examinations of TE patients

Lesion size greater than 7 cm was a strong predictor of respiratory failure, with an odds ratio (OR) of 3.827 (95% confidence interval [CI]: 0.482–30.365). Meanwhile, lung function testing revealed that the instantaneous expiratory flow in force of 50% vital capacity detection value was significantly reduced in the respiratory failure group, with a significant intergroup difference observed (*P* < .05). With regard to laboratory examination findings, patients with low hemoglobin levels were at higher risk of respiratory failure as compared to patients with normal hemoglobin levels. Among variables associated with arterial blood gas analysis, only PaCO_2_ level was associated with high respiratory failure risk, with patients with increased PaCO_2_ levels at significantly high risk for respiratory failure (OR: 3.529, 95% CI: 1.374–9.067).

### 3.4 TE patient surgical procedures

Surgery-associated risk factors are summarized in Table 3. An intraoperative bleeding volume greater than 1000 mL was a predictor of postoperative respiratory failure (OR: 5.647, 95% CI: 2.238–14.252). In contrast, factors of duration and type of surgery had no effect on risk of subsequent development of postoperative respiratory failure.

**Table 3:** Univariate analysis of surgical operations of TE patients stratified to the presence of respiratory failure.

Variables	Total (n = 139, %)	Nonrespiratory failure group (n = 113, %)	Respiratory failure group (n = 26, %)	OR (95% CI)	*P*-value
Operation time (h)					
≤4	51 (36.7)	44 (86.3)	7 (13.7)	Ref	
>4	88 (63.3)	70 (79.5)	18 (20.5)	1.616 (0.624–4.183)	.322
Type of surgery					
Thoracotomy	132 (95)	110 (83.3)	22 (16.7)	Ref	
Thoracoscope	7 (5.0)	4 (57.1)	3 (42.9)	3.750 (0.784–17.942)	.098
Bleeding volume (mL)					
≤1000	109 (78.4)	96 (88.1)	13 (11.9)	Ref	
>1000	30 (21.6)	17 (56.7)	13 (43.3)	5.647 (2.238–14.252)	<.001

CI = confidence interval, OR = odds ratio, TE = tuberculous empyema.

### 3.5 Multivariate analysis of risk factors for respiratory failure

Table 4 lists risk factors for respiratory failure of TE patients after surgery as determined using multivariate analysis. Among significant factors identified as associated with respiratory status, an intraoperative bleeding volume greater than 1000 mL had the highest OR, 6.452 (95% CI: 2.200–18.925). In addition, a pathologic preoperative pulmonary function test result showing a high PaCO_2_ level was an independent risk factor for respiratory failure, with an OR of 3.905 (95% CI: 1.276–11.947). The presence of TB lesion(s) in the contralateral lung was another respiratory failure-related risk factor that was revealed by multivariate analysis as significant, with an OR of 3.360 (95% CI: 1.208–99.350).

**Table 4:** Multivariate logistic regression of risk factors associated with the presence of respiratory failure after operation in TE patients.

Variables	Adjusted OR (95% CI)	*P*-value
TB lesion in contralateral lung	3.360 (1.208–9.350)	.020
Increased PaCO_2_	3.905 (1.276–11.947)	.017
Bleeding volume (mL)	6.452 (2.200–18.925)	.001

CI = confidence interval, OR = odds ratio, TE = tuberculous empyema.

## 4 Discussion

Postoperative respiratory failure is the most common complication that occurs in patients undergoing thoracic operations,^[10]^ with the reported incidence of respiratory failure after thoracic operations varying between 5% and 20%.^[15,16]^ Here our results revealed an incidence of respiratory failure of 18.7%, a higher incidence rate than numerous rates reported previously.^[17,18]^ In a large multicenter observational study by Arozullah and colleagues, respiratory failure developed in 3.4% of patients who had undergone noncardiac operations followed by postsurgical administration of mechanical ventilation of duration exceeding 48 hours.^[10]^ Therefore, variable incidence rates reported for postoperative respiratory failure reflect variability of definitions and procedures across studies.

This study confirmed several independent predictors of postoperative respiratory failure in TE patients. High intraoperative blood loss was the greatest risk factor for respiratory failure and had the highest OR value, with exudation of plasma components identified as the underlying hazard responsible for this high level of risk. Notably, high intraoperative blood loss is predominantly determined by thoracic cavity wound size. On the one hand, lesion size in TE patients is a major determinant of subsequent wound size. On the other hand, thoracic surgical procedures used for TE patients involve pleural decortication that supports optimized resolution of thickened parietal and visceral pleura. Meanwhile, the degree of pleural adhesion is another important contributing factor for increased postoperative drainage volume. Due to the fact that TE disease originates from local immunological responses that cause sustained stimulation of tubercle bacilli,^[19,20]^ prolonged TE disease tends to be associated with more serious pleural adhesions. Consequently, removal of such lesions depends on creation of wounds of greater size during surgery that, due to intimate proximity of TE lesions to lung tissues, leads to increased risk of postoperative respiratory failure.

Respiratory failure occurs when there is inadequate exchange of O_2_ and CO_2_ to meet metabolic needs.^[21]^ Here we found high preoperative PaCO_2_ level to be an independent predictor for respiratory failure in TE patients. A high PaCO_2_ level at baseline reflects retention of CO_2_ associated with insufficient ventilation of pulmonary tissues. In patients affected by TE, TE lesions protrude from the normal surrounding pulmonary tissues,^[20]^ leading to an increase in dead space that promotes greater CO_2_ retention. When TE patients undergo thoracic surgical procedures, the traumatized chest wall impairs the mechanics of ventilation, predisposing the TE patient to greater risk of respiratory failure. Thus, based on our experience, preoperative assessment of arterial blood gases is essential for identifying TE patients at high risk for postoperative respiratory failure.

Age has variably been reported to be a significant predictor of postoperative respiratory failure.^[15]^ Reddy and colleagues, after sorting patients into 3 age-based subgroups, clearly demonstrated that ORs for respiratory failure increased with advancing age.^[22]^ Nevertheless, results of this study do not support the premise that advancing age increases risk to patients of postsurgical respiratory failure.

Another risk factor associated with postsurgical respiratory failure, the presence of TB lesions in the contralateral lung (OR, 4.0) was detected after results were analyzed using a logistic regression model. As a respiratory infectious disease, MTB infection can lead to extensive lesion development that subsequently causes stiffening of the affected lung that shifts more and more pulmonary function burden to the contralateral lung. Meanwhile, coexistent lesions in the contralateral lung are a sign that dissemination of tubercle bacilli has occurred that is likely accompanied by erosion of lung segmentation regions. Breaching of the pleural interface, which occurs during thoracotomy and thoracoscopy, significantly decreases intrathoracic lung volume.^[23]^ Thus, due to surgically induced loss of lung volume, the contralateral lung must endure an even greater ventilation workload during surgery that leads to impaired contralateral lung capacity that may increase postoperative respiratory failure risk. In our previous studies of drug-resistant TB cases, use of effective anti-TB regimens during the postsurgical follow-up period played an important role in determining long-term patient outcomes after surgery^[24]^ by effectively preventing spread of tubercle bacilli to the nonsurgical lung. Therefore, our observations suggest that preoperative chemotherapy followed by surgical ablation of the TE lesion would reduce respiratory failure risk of patients afflicted with TB in the contralateral lung and improve survival.

We acknowledge several obvious limitations of this study. First, despite enrollment of TE cases over the past 2 decades, the observational single-institution retrospective study design may have limited the significance of the results obtained in this work. Second, the positivity grade of AFB smear results, which correlates with TB patient bacterial load, is a potential risk factor for postoperative respiratory failure. However, in this study only qualitative results, not the degree of positivity of AFB smear results, was determined by our hospital laboratory. Third, postoperative follow-up evaluations were not conducted of survivors of surgery who experienced respiratory failure, hampering further analysis to determine whether respiratory failure had a negative impact on survival. More specifically, the question that must be raised is whether acute respiratory failure is likely to develop into chronic respiratory failure, warranting further long-term follow-up monitoring of patients experiencing postoperative respiratory failure in order to answer this question. Finally, in spite of standardized training programs, levels of professional skills vary among thoracic surgeons as an additional potential confounding factor that was not investigated in our analysis.

## 5 Conclusion

In conclusion, our results demonstrate that respiratory failure is a predominant postsurgical complication experienced by TE patients. A high preoperative PaCO_2_ level and presence of TB lesion(s) in the contralateral lung are associated with increased patient risk of postoperative respiratory failure. Further clinical trials are urgently needed to identify appropriate interventions to prevent respiratory failure in high-risk patients.

## Author contributions

**Formal analysis:** Hongyun Ruan.

**Investigation:** Changfan Gong, Xinting Yang.

**Methodology:** FangChao Liu.

**Writing – original draft:** Hongyun Ruan.

**Writing – review & editing:** Xinting Yang, Ming Han.
